# Soybean cyclophilin GmCYP1 interacts with an isoflavonoid regulator GmMYB176

**DOI:** 10.1038/srep39550

**Published:** 2017-01-11

**Authors:** Hemanta Raj Mainali, Arun Kumaran Anguraj Vadivel, Xuyan Li, Mark Gijzen, Sangeeta Dhaubhadel

**Affiliations:** 1 Department of Biology, University of Western Ontario, 1151 Richmond St, London, ON, N6A 5B7, Canada; 2 London Research and Development Centre, Agriculture and Agri-Food Canada, 1391 Sandford Street, London, ON, N5V 4T3, Canada; 3 College of Plant Science, Jilin University, Changchun, 130062, China

## Abstract

Cyclophilins (CYPs) belong to the immunophilin superfamily with peptidyl-prolyl *cis-trans* isomerase (PPIase) activity. They catalyze the interconversion of the *cis-* and *trans-*rotamers of the peptidyl-prolyl amide bond of peptides. A yeast-two-hybrid screening using the isoflavonoid regulator GmMYB176 as bait identified GmCYP1 as one of the interacting proteins in soybean embryos. GmCYP1 localizes both in the nucleus and cytoplasm, and interacts *in planta* with GmMYB176, in the nucleus, and with SGF14l (a soybean 14-3-3 protein) in the nucleus and the cytoplasm. GmCYP1 contains a single cyclophilin-like domain and displays a high sequence identity with other plant CYPs that are known to have stress-specific function. Tissue-specific expression of *GmCYP1* revealed higher expression in developing seeds compared to other vegetative tissues, suggesting their seed-specific role. Furthermore, *GmCYP1* transcript level was reduced in response to stress. Since isoflavonoids are involved in plant stress resistance against biotic and abiotic factors, the interaction of GmCYP1 with the isoflavonoid regulators GmMYB176 and 14-3-3 protein suggests its role in defense in soybean.

Cyclophilins (CYPs) are a group of proteins that possess peptidyl prolyl *cis/trans* isomerase (PPIase) activity. They are involved in protein folding by interconverting the *cis-* and *trans-*rotamers of the peptidyl prolyl amide bond of peptides, and are broadly classified into three major classes: parvulins, FK506 binding proteins (FKBP) and cyclophilins^1^. FKBP and CYPs are collectively called immunophilins as they were originally identified as receptors for immunosuppressive drugs, FK506 and cyclosporine A, respectively^2^,^3^,^4^. CYPs are present in a wide range of organisms, from archaea, bacteria to plants and animals^5^,^6^,^7^. Genome-wide analyses of *CYP* genes in various organisms revealed disparity in the number of genes, ranging from 8 to 16 in organisms such as *Drosophila*^8^, *Caenorhabditis elegans*^9^, *Saccharomyces cerevisiae*^10^, and human^8^. A large number of studies focussed on human CYP, hCYPA, have shown its crucial role in protein folding, signal transduction, cell signaling, regulation of gene expression, immune response, and disease conditions^11^,^12^,^13^.

The first plant *CYPs* were identified concomitantly in 1990, from tomato (*Lycopersicon esculentum*), maize (*Zea mays*), and oilseed rape (*Brassica napus*)^14^. With the advancement in genome sequencing technology, and availability of plant genome sequence data in public domain, the identification and characterization of plant *CYPs* has progressed significantly in the recent years. Compared to other organisms, photosynthetic organisms contain significantly higher number of CYPs; 62 in soybean^15^, 35 in *Arabidopsis*^16^,^17^, 28 in rice^17^,^18^, and 26 in *Chlamydomonas*^19^.

In order to combat biotic and abiotic stress, sessile organisms like plants have developed several sophisticated mechanisms at the cellular and molecular levels^20^,^21^. One of the consequences of abiotic stress is the denaturation and aggregation of cellular proteins leading to cell death. The chaperone-like activity of CYPs and their role in the rate-limiting step of protein folding by peptidyl prolyl bond isomerization^22^ is associated with their involvement in stress responses. Expression of many plant *CYPs* is induced in response to stress suggesting their possible function in stress tolerance. For example, expression of the *Arabidopsis CYP, ROTAMASE CYCLOPHILIN 1 (ROC1),* increases upon wounding^23^. Similarly, maize and bean *CYP* gene expression increases in response to heat stress, wounding, high salinity, or low temperature^24^. *Solanum commersonii CYP* gene expression is also up-regulated by low temperature, abscisic acid, drought, or wounding^25^. Pepper *CYPs* are differentially regulated during abiotic stress and pathogen infection^26^. Ectopic expression of *Thellungiella halophile CYP, ThCYP1,* in fission yeast and tobacco cells increased salt tolerance^27^. Transgenic *Arabidopsis* plants overexpressing pigeon pea *CYP (CcCYP1*) showed enhanced PPIase activity under stressed conditions, which correlated with their increased tolerance against drought, salinity and high temperature^28^. Similarly, overexpression of cotton *CYP (GhCYP1*) in tobacco plants conferred tolerance against salt stress and fire-blight disease^29^. Together, these findings clearly demonstrate a role for plant CYPs in stress tolerance.

Soybean (*Glycine max*) is a grain legume belonging to the family *Fabaceae*. Soybean seeds provide a major supply of oil, protein and beneficial plant natural compounds such as isoflavonoids and saponins. The soybean genome contains 88,647 predicted transcripts and 56,044 protein coding loci located on 20 different chromosomes^30^. Previously, we performed a genome-wide analysis of soybean *CYPs* and identified 62 *CYP* genes^15^. Among these, GmCYP1 has been shown to act as a “helper” to *Phytophthora sojae* RXLR effector Avr3b by activating its hydrolase activity in plant cells^31^. The protein-protein interaction between GmCYP1 and Avr3b was shown to be isoform-specific since GmCYP1 paralogs failed to interact with Avr3b. Here we present a molecular characterization of *GmCYP1* covering its sequence analysis, phylogeny, temporal and spatial expression, subcellular localization, and provide the evidence for its possible role in isoflavonoid biosynthesis and stress response in soybean.

## Results and Discussion

### Isolation, sequence analysis and phylogeny of GmCYP1

GmCYP1 was identified in our Y2H screening as a protein that demonstrated protein-protein interaction with the isoflavonoid regulator GmMYB176. The Y2H assay was performed to identify GmMYB176-interacting proteins using GmMYB176 as the bait protein and proteins from soybean embryos (50–60 days after pollination) as prey. Of the several hundred yeast colonies screened, 6.5% contained a sequence corresponding to *GmCYP1* (accession #AF456323, locus Glyma.11G098700). *GmCYP1* is predicted to contain only one exon (519 bp), and is located on the long arm (q arm) of chromosome 11, approximately 16 Mb from the centromere. It encodes a single domain protein of 172 amino acid residues with a calculated molecular mass of 18.22 kDa and a pI of 8.69. The cyclophilin-like domain in GmCYP1 is predicted in between the amino acid residues 7 and 169.

**Figure 1:**
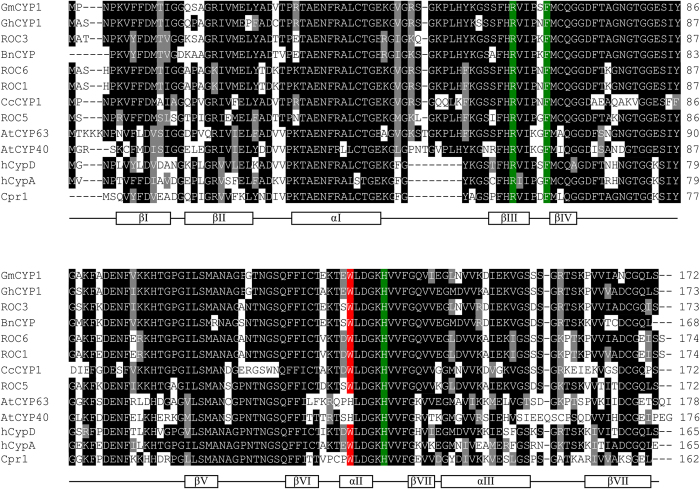
Multiple sequence alignment of deduced amino acid sequence of GmCYP1 with CYPs from other species. Amino acid sequences of ROC3, BnCYP, GhCYP1, CcCYP1, ROC6, ROC1, ROC5, Cpr1, hCYP-D, hCYP-A, AtCYP40, AtCYP63 and GmCYP1 (refer to Table S1 for accession numbers) were aligned by ClustalW, and imported into BOXSHADE 3.21 for shading. Identical amino acids are shown in the dark box and similar amino acids are indicated by the grey box. Amino acid residues involved in PPIase activity (R_55_, F_60_ and H_126_) (Zydowsky *et al*.^32^) and CsA binding (W_121_) (Liu *et al*.^33^; Zydowsky *et al*.^32^) are highlighted with green and red, respectively. Secondary structure is shown below the alignment. The relative positions of amino acids indicated for PPIase activity and CsA binding sites, and the secondary structure features are based on hCYP-A (Kallen *et al*., 1991).

In order to find sequences closely related to GmCYP1, a protein-protein BLAST (BLASTP) was performed using GmCYP1 as a query against the NCBI non-redundant protein database. A list of 12 high-scoring and previously characterized CYPs is shown in Table S1. Alignment of the deduced sequence of GmCYP1 with previously characterised CYPs from several different plant species, human, yeast, and two *Arabidopsis* multi-domain CYPs (AtCYP40 and AtCYP63) revealed two general features (Fig. 1). First, three amino acid residues that critically affect PPIase activity (R_55_, F_60_ and H_126_)^32^ are conserved in all CYPs aligned. Second, the tryptophan residue (W_121_) implicated in substrate cyclosporinA binding^32^,^33^ is present in all of the CYPs studied except in the multi domain CYPs.

**Figure 2:**
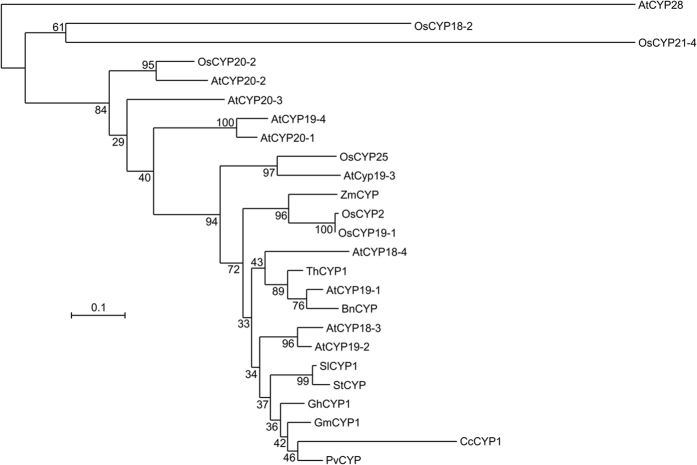
Phylogenetic analysis of GmCYP1 with other known plant CYPs. Deduced amino acid sequence of GmCYP1 was aligned with other plant CYPs using Clustal omega, and the alignment was imported to MEGA 6.0 to create a phylogenetic tree using Maximum Likelihood method. Numbers indicate bootstrap value in percentage. The tree was exported into Interactive Tree of Life (http://itol.embl.de/) for annotation and manipulation. Accession numbers used for the alignment are: OsCYP2 (AAA57046, *O. sativa*), OsCYP20-2 (XP_015640756, *O. sativa*), OsCYP18-2 (XP_015649749, *O. sativa*), OsCYP25 (XP_015610625, *O. sativa*), OsCYP21-4 (XP_015647974, *O. sativa*), AtCYP19-3 (NP_191166, *A. thaliana*), AtCYP18-3 (NP_195585, *A. thaliana*), AtCYP18-4 (NP_195213, *A. thaliana*), AtCYP19-1 (NP_179251, *A. thaliana*), AtCYP19-2 (NP_179709, *A. thaliana*), AtCYP19-4 (NP_180557, *A. thaliana*), AtCYP20-1 (NP_191166, *A. thaliana*), AtCYP20-2 (NP_196816, *A. thaliana*), AtCYP20-3 (NP_001154684, *A. thaliana*), AtCYP28 (NP_198360, *A. thaliana*), AtCYP37 (NP_188171, *A. thaliana*), AtCYP38 (NP_186797, *A. thaliana*), AtCYP40 (NP_565381, *A. thaliana*), GhCYP1 (ACT63839, *G. hirsutum*), SlCYP1 (AAA63543, *S. lycopersicum*), BnCYP (AAA62706, *B. napus*), ZmCYP (CAA48638, *Z. mays*), CcCYP1 (ADB04247, *C. cajan*), ThCYP1 (AAR27291, *T. halophila*), StCYP (AAD22975, *S.tuberosum*), PvCYP (CAA52414, *P. vulgaris*).

A phylogenetic analysis of GmCYP1 and other functionally characterized plant CYPs clustered GmCYP1 close to *GhCYP1, CcCYP1* and *PvCYP* from cotton, pigeon pea and common bean, respectively. Both *GhCYP1* and *CcCYP1* are known to have stress-specific function (Fig. 2). Overexpression of *GhCYP1* in tobacco conferred increased tolerance to biotic and abiotic stress^29^. Similarly, *Arabidopsis* plants overexpressing *CcCYP1* showed higher PPIase activity during stress and increased tolerance against multiple abiotic stresses as compared to control^28^. Differential accumulation of *PvCYP* transcripts in response to various external stimuli suggested that it may possess a stress-related function^24^. The high amino acid sequence identity of GmCYP1 with GhCYP1 (96%), CcCYP1 (81%) and PvCYP (96%), suggests similar possible functions of GmCYP1 in stress response.

### GmCYP1 localizes in the nucleus and the cytoplasm

**Figure 3:**
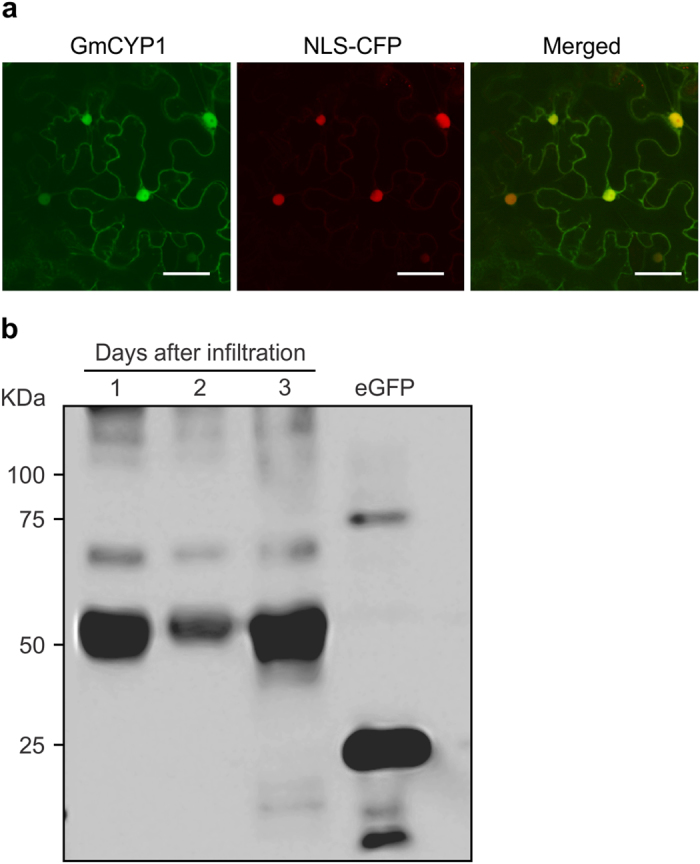
Subcellular localization of GmCYP1. *A. tumefaciens* GV3101 carrying the plasmids with GmCYP1-YFP and nuclear localizing CFP (NLS-CFP) constructs were co-infiltrated into *N. benthamiana* leaves and visualized by confocal microscopy. Expression of **(a)** GmCYP1-YFP, **(b)** NLS-CFP, **(c)** images of A and B merged to confirm nuclear localization of GmCYP1. Scale bars indicate 50 μm. **(d)** Western blot analysis of translational fusion of GmCYP1-YFP proteins. GmCYP1-YFP was transiently expressed in *N. benthamiana* leaves for 1, 2 or 3 days, and protein accumulation was measured. Proteins (30 μg) were separated on a SDS-PAGE and transferred to PVDF membrane by electroblotting. GmCYP1-YFP protein was detected by sequential incubation of the blot with anti-GFP antibody and anti-mouse IgG conjugated with HRP, followed by chemiluminescent reaction. eGFP is shown as a positive control.

To further study the subcellular localization of GmCYP1, a translation fusion of GmCYP1 with YFP was created under the control of 35S promoter, and transiently expressed in *N. benthamiana* leaves. Although there was no predicted nuclear localization sequence in GmCYP1, confocal imaging of the GmCYP1-YFP-infiltrated tobacco leaves showed both nuclear and cytoplasmic localization (Fig. 3a). Nuclear localization of GmCYP1 was confirmed by co-expression of GmCYP1-YFP and NLS-CFP.

Molecules of size smaller than 20–40 kDa, such as ions, water, and small proteins, can pass through the nuclear pore complex by diffusion^34^, whereas movement of larger molecules (70 kDa or higher) entails an active transport system^35^, mediated by transport receptors and signal peptides^36^. The size of GmCYP1-YFP (45.22 kDa) is not considerably larger than the size of molecules that can pass through the nuclear pore complex by diffusion. It is also possible that heterologous protein GmCYP1-YFP, expressed in *N. benthamiana* is cleaved by the endogenous host proteases and only the cleaved YFP fragments are localized to the nucleus. The confirmation of the YFP signal in the nucleus arising from the intact GmCYP1-YFP and not from the cleaved product of a fusion protein was performed by Western blot analysis (Fig. 3b). Therefore, it is not clear whether GmCYP1-YFP localization in the nucleus was due to passive diffusion or to active transport. Regardless, its nuclear localization indicates a possible role in the regulation of gene expression.

### GmCYP1 interacts with GmMYB176 *in planta*

**Figure 4:**
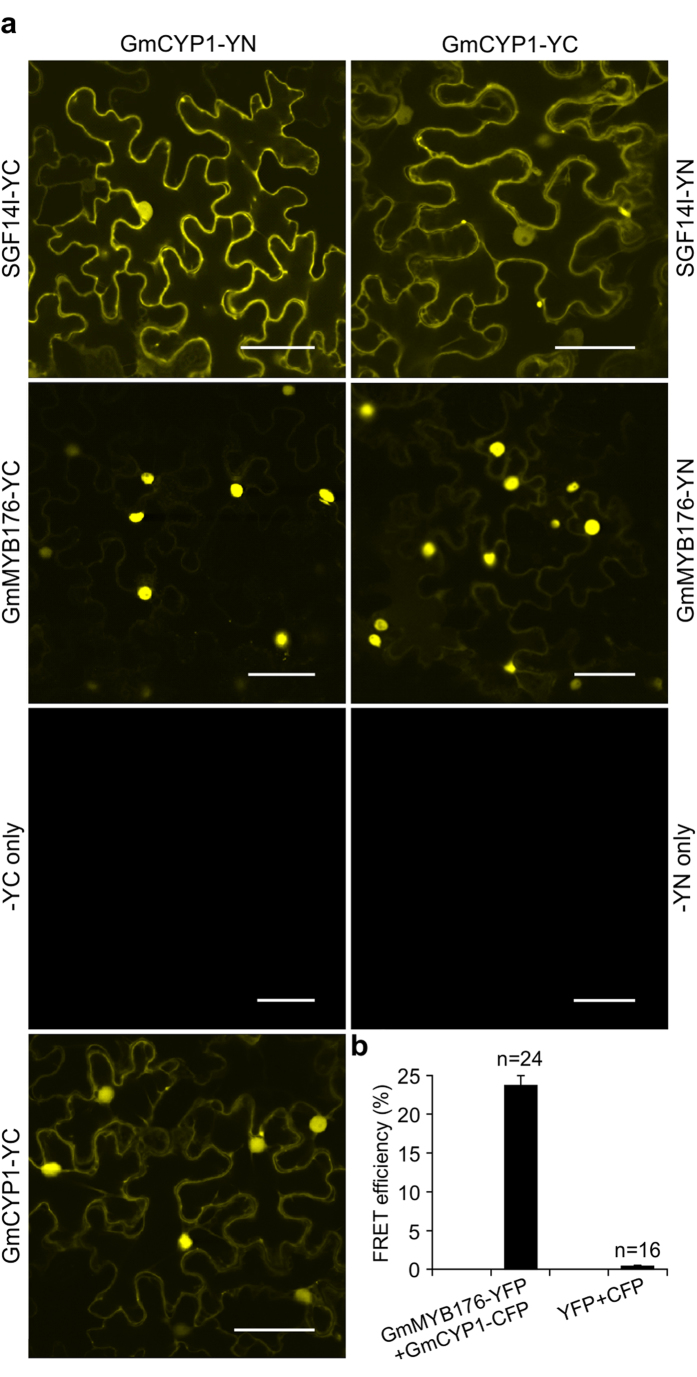
GmCYP1 interacts with GmMYB176 and SGF14l *in planta*. *N. benthamiana* leaves were co-transformed with *A. tumefaciens* carrying **(a)** GmCYP1-YN and SGF14l-YC or their reciprocal combination, GmCYP1-YN and GmMYB176-YC or their reciprocal combination, GmCYP1-YN and GmCYP1-YC with vector only control (-YN or –YC only), GmCYP1-YN and GmCYP1-YC, and observed by confocal microscopy. Protein-protein interactions were visualized by a strong yellow fluorescence. Scale bars indicate 50 μm. **(b)** FRET analysis demonstrating protein-protein interactions between GmMYB176 and GmCYP1. The CFP and YFP channels were excited with 458 nm and 514 nm lasers respectively, and FRET efficiencies were calculated in multiple samples (n > 15). The empty pEG101 (YFP) and pEG102 (CFP) vector pairs were used as a FRET signal control.

GmCYP1 was identified as one of the interacting proteins of GmMYB176 in the Y2H assay. Protein chaperones often bind with misfolded bait proteins when a protein is overexpressed or heterologously expressed. To confirm that GmCYP1 is a true GmMYB176-interacting protein, we performed a targeted Y2H assay using GmCYP1 and GmMYB176. However, our targeted Y2H assay failed to verify the interaction between GmCYP1 with GmMYB176. This result led us to hypothesize that there may be an indirect interaction between GmCYP1 and GmMYB176 *via* involvement of protein (s) that may not be conserved between the species. Therefore, a BiFC assay^37^ was carried to further investigate *in planta* interaction between GmCYP1 and GmMYB176. Translational fusions of GmMYB176 and GmCYP1 were created with N-terminal (YN) or C-terminal (YC) halves of YFP^38^ and co-expressed in *N. benthamiana* leaf epidermal cells in the following combinations: (A) GmCYP1-YN and GmMYB176-YC, (B) GmMYB176-YN and GmCYP1-YC. The negative controls used for the experiment were co-expression of GmCYP1-YN and -YC only or GmCYP1-YC and -YN only. As shown in Fig. 4a, GmCYP1 interacts with GmMYB176 *in planta*, and the interaction between GmCYP1 and GmMYB176 was strong in the nucleus. Similar results were obtained for the reciprocal combinations. No YFP signal was detected during co-expression of control constructs -YC or -YN with GmCYP1-YN or GmCYP1-YC, respectively, confirming that YFP signal was due to the protein-protein interaction *in planta.* Furthermore, we measured the strength of interaction by using FRET approach. It is a powerful tool for non-invasive monitoring of protein-protein interactions that involves transfer of energy between two closely positioned fluorophores^39^, and has been widely used to determine the interactions between proteins in living plant cells^40^,^41^,^42^. Translational fusions of GmMYB176 with YFP and GmCYP1 with CFP were co-expressed in *N. benthamiana* leaf epidermal cells and analysed for FRET efficiency between the reporters *in vivo*. The results revealed a FRET efficiency of 23.8%, indicating a close proximity of GmMYB176 and GmCYP1 and their co-existence in a complex (Fig. 4b). Empty vectors containing YFP only and CFP only were used as negative control which showed a FRET efficiency of 0.5%. A FRET value of 8–10% was considered as the background level in previous studies^43^,^44^.

When *A. tumefaciens* GV3101 strains carrying BiFC plasmids containing GmCYP1-YN or GmCYP1-YC were co-infiltrated into *N. benthamiana* leaves and visualized by confocal microscope, a strong yellow fluorescence was observed in the nucleus and relatively weaker fluorescence in the cytoplasm, suggesting that GmCYP1 forms a homodimer in both cellular compartments *in planta* (Fig. 4a). Even though recombinant hCYP-A has been reported to form monomers, dimers, and trimers when expressed in *E. coli*^45^, there is no published literature on homo-dimerization of plant CYPs. Search for predicted motifs in GmCYP1 identified a putative phosphorylation (pST binding) site within the GmCYP1 sequence (97-ENFVKKH**T**GPGILSM-112), where T_105_ is potentially phosphorylated. The pST binding motifs are binding sites for 14-3-3 family of proteins. 14-3-3 protein functions as a dimer to bind with its client proteins where each monomer in the dimer is capable of interacting with a separate client protein. The dimeric nature of 14-3-3 proteins allows them to serve as scaffolds by bringing two regions of the same proteins into proximity or two different proteins together^46^. We have previously demonstrated that GmMYB176 interacts with 14-3-3 proteins, thereby affecting its subcellular localization^38^. The interactions between GmCYP1 and GmMYB176 in the Y2H screen and in BiFC assay where soybean and *N. benthamiana* 14-3-3 s possibly bring the two proteins together could be explained if GmCYP1 is a true client of 14-3-3 protein. Therefore, we performed a BiFC assay between SGF14l (a soybean 14-3-3) and GmCYP1. Indeed, GmCYP1 interacted with SGF14l *in planta* (Fig. 4), suggesting that 14-3-3 proteins may act as a scaffold to facilitate binding of GmCYP1 and GmMYB176. Despite that the mechanism and consequence of GmCYP1 dimerization are not yet known, it is possible that binding of 14-3-3 with GmCYP1 could bring two GmCYP1 monomers together to produce fluorescence in the BiFC assay. Further, it is not clear whether the binding of GmCYP1 and GmMYB176 mediated by SGF14l is involved in the *CHS8* gene regulation, and subsequent isoflavonoid biosynthesis in soybean, or to some other, as-yet unknown function.

### *GmCYP1* is expressed ubiquitously in soybean tissues

**Figure 5:**
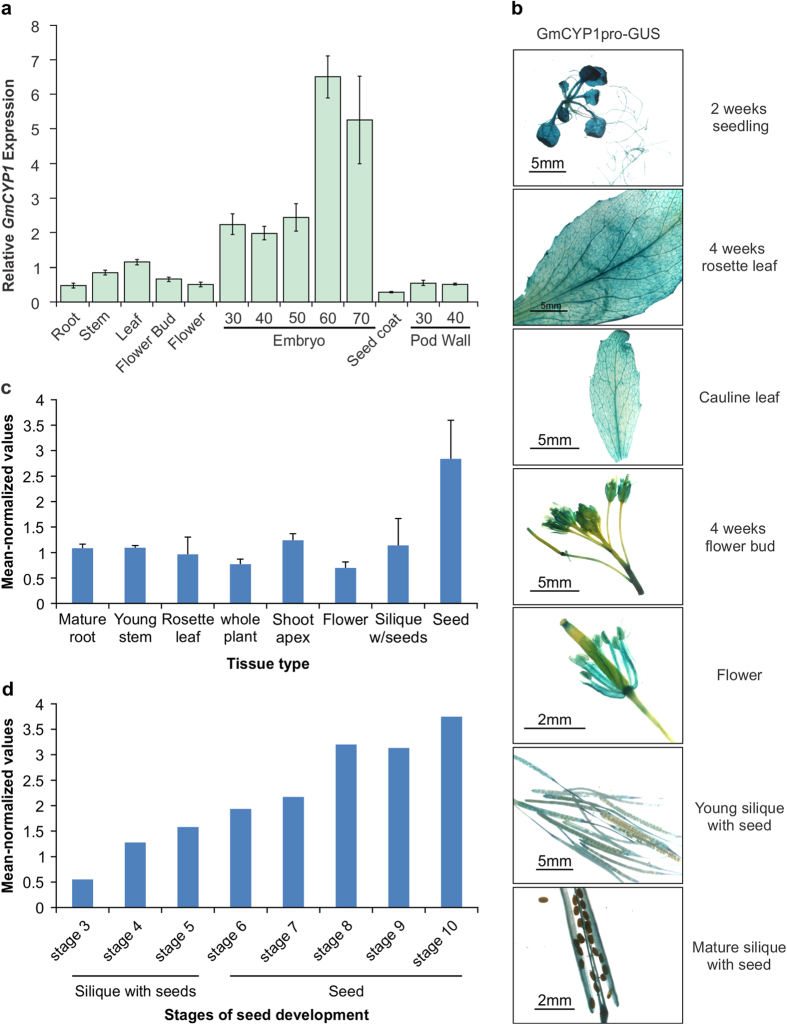
Expression analysis of *GmCYP1* in soybean. **(a)** Total RNA extracted from soybean root, stem, leaf, flower bud, flower, embryo (30, 40, 50, 60, and 70 days after pollination), seed coat and pod wall (30 and 40 days after pollination) were used for quantitative RT-PCR analysis of *GmCYP1*. Two biological replicates and three technical replicates for each biological replicate were carried out. The standard error of the mean is represented by an error bar. The data were normalized against *SUBI-3* gene. **(b)** Histochemical analysis of GmCYP1promoter-GUS activity in vegetative and reproductive tissues during various stages of development in *Arabidopsis*. Construct containing a *GmCYP1* promoter driven *GUS* gene was transformed into *Arabidopsis* and selected T2 transgenic plants were used for analysis. **(c)** The mean normalized expression values of *ROC1* in different *Arabidopsis* tissues and **(d)** stages of seed development were obtained from AtGenExpress Visualization Tool (http://jsp.weigelworld.org/expviz/expviz.jsp). Error bars indicate the standard deviation of the mean. The stages of seed developments are: stage 3, mid globular to early heart embryos; stage 4, early to late heart embryos; stage 5, late heart to mid torpedo embryos; stage 6, mid to late torpedo embryos; stage 7, late torpedo to early walking-stick embryos; stage 8, walking-stick to early curled cotyledons embryos; stage 9, curled cotyledons to early green cotyledon embryos; and stage 10, green cotyledon embryos.

To study the temporal and spatial expression of *GmCYP1* in soybean, a detailed transcript analysis using quantitative PCR was performed. Total RNA isolated from different tissues of soybean cultivar Harosoy63, at several different developing stages, was used in the analysis. As shown in Fig. 5a, *GmCYP1* was expressed in all soybean tissues, albeit at various levels. Transcript accumulation was higher in embryos compared to that in other tissues. The level of *GmCYP1* transcript increased in soybean embryos during the late developmental stages, showing highest levels (a 3-fold increase) in the embryos at 60 and 70 days after pollination compared to that in embryos at 30, 40 or 50 days after pollination (mid developmental stage) (Fig. 5a). No *GmCYP1* transcripts were detected at early embryo developmental stages or in mature seeds^15^. Seed coat tissues accumulated 23- and 18-fold less *GmCYP1* transcripts compared to the embryos at 60 and 70 days after pollination, respectively. The seed coat is rich in defensive and pathogen-related proteins^47^. Among all the soybean tissues and organs tested for the expression of *GmCYP1*, the level of expression was lowest in the seed coat.

For an in-depth analysis of *GmCYP1* expression, we constructed a reporter vector containing the 1,148 bp *GmCYP1* promoter fragment (upstream of the translation start site) to drive *GUS* gene expression. *Agrobacterium* strain containing the p*GmCYP1*pro*-GUS* was transformed into wild type *Arabidopsis*. Transgenic lines (8–10 independent T_2_ progenies) carrying *GmCYP1*pro*-*GUS were selected for measuring GUS activity in different tissues during development. As observed in soybean tissues, the *GmCYP1* promoter was active in most tissues of *Arabidopsis* (Fig. 5b). Strong GUS activity was observed in leaves and roots of young seedling while relatively less activity was found in the hypocotyl. This study revealed additional information on tissue-specific expression of *GmCYP1*. For example, in leaves, GUS staining was more pronounced in the vascular tissues. Similarly, *GmCYP1* promoter was active in flower buds, stamen, stigma and silique walls but not in flower stem, style, petal or seeds. Failure to observe GUS activity in seeds could be due to weaker activity of the *GmCYP1* promoter in the seed coat, and is supported by *GmCYP1* expression in seed coat as shown in Fig. 5a. Similar results were observed for soybean *chalcone synthase (CHS)* gene promoters. Both *CHS7* and *CHS8* promoter driven GUS activities were absent in *Arabidopsis* seeds, despite the fact that *CHS7* and *CHS8* transcripts were present in the seed coat in soybean^48^. It is possible that the observed differences of gene expression in soybean and *Arabidopsis* may be caused by the presence or absence of the required regulatory factors in the specific tissue or developmental stage. Like *GmCYP1,* the *Arabidopsis* ortholog of *GmCYP1, ROC1* (AGI:At4g38740), also exhibited higher transcript accumulation in seeds than in other tissues (Fig. 5c). The normalized mean expression data of *ROC1* was compiled from AtGenExpress Visualization Tool (http://jsp.weigelworld.org/expviz/expviz.jsp). The expression of *ROC1* increased gradually, and approx. 7-fold throughout seed development, from a relatively low value (0.55) at the mid globular stage (stage 3), to values of 3.2–3.75 during the later developmental stages (early curled cotyledon embryos, stage 8) to green cotyledon embryos (stage 10) (Fig. 5d). The similar expression pattern during embryo development of *GmCYP1* and its *Arabidopsis* ortholog *ROC1* suggests a conserved role for *GmCYP1* and *ROC1* in seed development.

**Figure 6:**
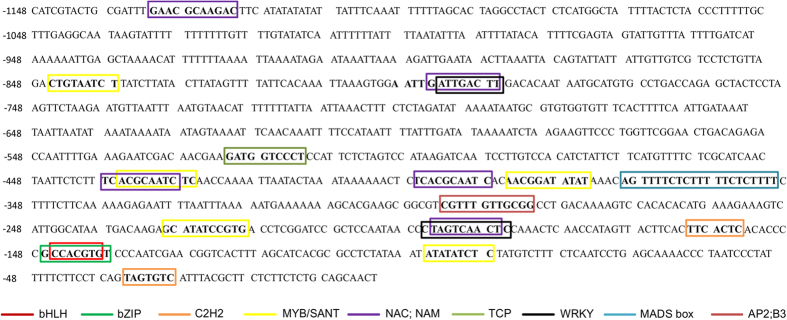
Promoter analysis of *GmCYP1*. A 1148 bp upstream of translation start site of *GmCYP1* was used for analysis using PlantPan2.0 database (http://plantpan2.itps.ncku.edu.tw/promoter.php). The transcription factors that are known for their role in stress and hormonal pathways are indicated.

To identify putative *cis*-elements that regulate the expression of *GmCYP1* gene in soybean, we performed *in silico* motif analysis of 1148 bp upstream of translational start site using PlantPan2.0 database (http://plantpan2.itps.ncku.edu.tw/promoter.php). The transcription factors specific to soybean was selected during the analysis. Transcription factors that are known for their role in stress and hormonal pathways are shown in Fig. 6. The analysis identified several sequence motifs that are recognized by a number of key factors such as bZIP, MYB, WRKY, bHLH, AP2, NAC factors have been identified. Besides their role in normal plant growth and development, the stress-specific roles of these factors have been well documented in crop plant and model species^49^,^50^,^51^.

### *GmCYP1* expression is reduced in response to stress

**Figure 7:**
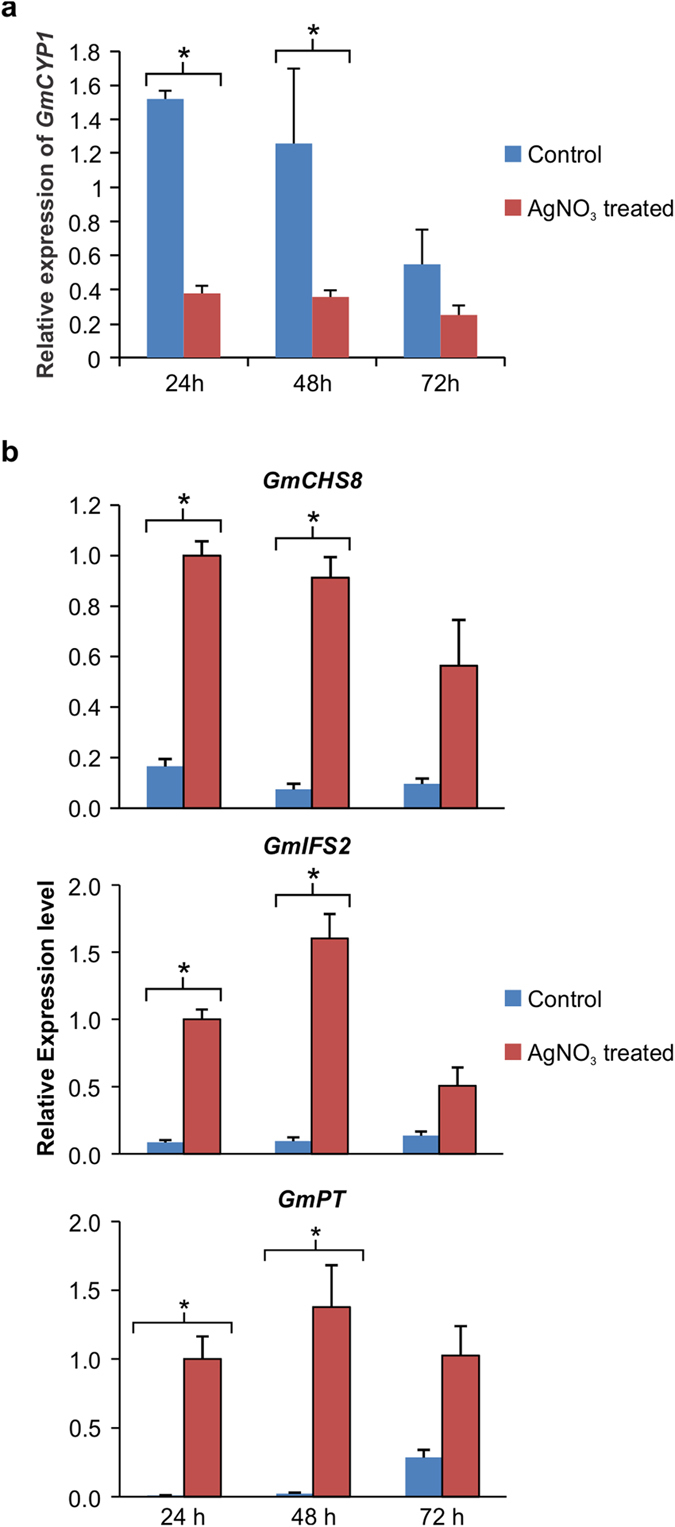
Expression of *GmCYP1* and isoflavonoid biosynthetic genes in response to stress. Etiolated soybean hypocotyls were treated with either 1 mM AgNO_3_ or water (control) for 24, 48 or 72 h, and tissues were used to evaluate **(a)** *GmCYP1* **(b)** isoflavonoid biosynthetic gene (*GmCHS8, GmCHI1B1, GmIFS2, GmPT*) transcript accumulation using quantitative RT-PCR. Error bars indicate SEM of two biological replicates and three technical replicates for each biological replicate. The data were normalized against the *SUBI-3* gene for *GmCYP1* and *CON4* gene for isoflavonoid genes. Asterisk (*) indicate significant difference between the samples using Student’s *t*-test.

*P. sojae* effector Avr3b contains Nudix hydrolase activity *in planta* that is required for the virulence of the pathogen in soybean^52^. Recently, it has been shown that GmCYP1 acts as a ‘helper’ by directly interacting with Avr3b, and modulating the hydrolase activity of it in soybean^31^. This GmCYP1-Avr3b interaction is required for the virulence and avirulence functions of Avr3b in soybean. Treatment of soybean hypocotyls with AgNO_3_ is known to induce defense response and phytoalexin accumulation^53^. Here we measured the accumulation of *GmCYP1* transcripts in response to stress by treating etiolated soybean hypocotyls with AgNO_3_ and monitoring *GmCYP1* expression 24, 48 or 72 h post-treatment. The results revealed that AgNO_3_ treated soybean hypocotyls accumulate reduced level of *GmCYP1* transcripts compared to the control hypocotyls at all the time points under the study (Fig. 7a). The difference in the level of *GmCYP1* transcript accumulation between AgNO_3_ treated and control hypocotyls was more pronounced at 24 h compared to 48 or 72 h post-treatment.

Isoflavonoid phytoalexins are host-produced antimicrobial compounds that are massively induced by pathogen attack or any other stress^54^,^55^. GmMYB176 regulates isoflavonoid biosynthesis by regulating *CHS8* gene expression^56^. Since GmCYP1 interacts with GmMYB176, and its expression is down-regulated upon stress, whereas phytoalexin biosynthesis is induced upon stress, we measured the expression levels of isoflavonoid biosynthetic genes *GmCHS8, isoflavone synthase (GmIFS2)* and a isoflavonoid-specific *prenyltransferase (GmPT*) in response to AgNO_3_ treatment. Our results revealed that expression of all these genes were induced upon AgNO_3_ treatment albeit at different levels (Fig. 7b). GmCHS8 is the first enzyme in the flavonoid pathway, and as compared to control, its transcripts were accumulated at 6, 12 and 5.8 fold higher at 24, 48 and 72 hours, respectively after AgNO_3_ treatment. GmIFS2 is a key legume-specific enzyme that introduces the isoflavonoid branch in the flavonoid pathway in legumes. Transcript levels of *GmIFS2* were 11.6, 17 and 3.7 fold higher after 24, 48 and 72 hours, respectively in AgNO_3_ treated samples compared to control. To confirm if downstream phytoalexin biosynthetic genes are induced upon stress, we measured the transcript levels of *GmPT*. Our results demonstrated that *GmPT* transcripts accumulated at 121, 64 and 3.5 fold greater than control at 24, 48 and 72 hours, respectively after AgNO_3_ treatment (Fig. 7b). A significantly higher difference in the expression of isoflavonoid genes were observed only when there was a significant reduction of *GmCYP1* gene expression (Fig. 7).

Several studies have shown that pathogen effectors interact with plant helper proteins for their activation and proper function^57^,^58^. Activated effector proteins bind with plant targets to suppress plant defenses and otherwise enable pathogen growth. For effectors encoded by *Avr* genes, activation can also result in detection by an immune receptor encoded by a resistance (*R*) gene. Thus, activation of Avr3b by GmCYP1 triggers immunity in soybean cultivars containing Rps3b, whereas activation of Avr3b in soybean cultivars lacking Rps3b enables pathogen growth^31^. Unlike many other plant CYPs that function in protecting plants during biotic and abiotic stress^29^,^59^, GmCYP1 acts as a susceptibility factor in soybean, at least in instances when Avr3b does not trigger immunity^31^. The expression of susceptibility proteins is generally induced during infection in susceptible plants^60^. However, our results show that *GmCYP1* transcripts are reduced in stressed plants compared to controls. This finding, together with the results that show GmCYP1 interacts with GmMYB176, suggests a role for GmCYP1 as a negative regulator of plant defense and isoflavonoid biosynthesis in soybean. The high expression of GmCYP1 in the late stage developing embryos cannot be explained by this hypothesis because isoflavonoid biosynthesis is active in seed tissues, albeit this is seed isoflavonoid and not phytoalexin isoflavonoid biosynthesis.

Overall, this study presents a detailed analysis of *GmCYP1*. The presence of predicted cyclophilin domain, subcellular localization and its sequence homology with other identified CYPs from other organisms provides insights into its putative function. The interaction of GmCYP1 and GmMYB176 is particularly intriguing because of the conditional functionality of GmCYP1 in effector-triggered immunity or susceptibility to the pathogen *P. sojae*. It seems more than coincidence that the biosynthesis of isoflavonoid phytoalexins, being necessary for resistance to *P. sojae*, is also connected to GmCYP1. Since isoflavonoids are involved in plant stress resistance against biotic and abiotic factors^61^,^62^,^63^, the interaction of GmCYP1 with isoflavonoid regulators and its potential role as a suppressor of plant defense merits further investigation.

## Methods

### Plant growth conditions

Soybean (*Glycine max* [L.] Merr) cultivar Harosoy 63 was grown in AAFC-London field plots during 2011 and 2012 for tissue collection. *Nicotiana benthamiana* plants were grown in pots under 16 h light at 25 °C and 8 h dark 20 °C cycle with 70–80% relative humidity and 100–150 μmol m^2^/s light intensity.

### RNA extraction and quantitative RT-PCR analysis

Total RNA was extracted from soybean tissues according to Wang and Vodkin^64^. The RNA samples were quantified using a NanoDrop spectrophotometer (Thermo Scientific, USA), and their integrity was checked. Total RNA (1 μg) from each sample was used for cDNA synthesis using the QuantiTect® Reverse Transcription Kit (Qiagen, USA). For quantitative RT-PCR, SsoFast^TM^ EvaGreen® Supermix (Bio-Rad, USA) was used with the CFX96 real-time PCR detection system (Bio-Rad, USA). Quantitative analysis of *GmCYP1* and isoflavonoid biosynthetic gene expression was carried out using the primers listed in Table S2. The amplicons were cloned into a pGEM-T Easy vector (Promega, USA), and its sequence verified. *SOYBEAN UBIQUITIN-3 (SUBI-3*) or *CON4* was used as a reference gene for data normalization and to calculate the relative mRNA levels. The data were analyzed using CFX manager (Bio-Rad, USA).

### Plasmid constructions

Full length *GmCYP1* was amplified from soybean cDNA constructed from mature embryo (60 and 70 DAP) using the primers GmCYP1-Gate-F: 5′- GGGGACAAGTTTGTACAAAAAAGCAGGCTTCATGCCTAACCCTAAGGTCTTCTTC-3′ and GmCYP1-Gate-R: 5′-GGGGACCACTTTGTACAAGAAAGCTGGGTCAGAGGGTTGACCGCAGTTG- 3′. The PCR product was recombined into pDONR-Zeo (Invitrogen, USA) using the BP clonase reaction mix (Invitrogen, USA), transformed into *Escherichia coli* DH5α, and grown on LB media supplemented with zeocin (50 μg/mL). The *E. coli* colonies containing recombinant plasmids were screened by colony PCR using gene-specific primers to identify pDONR-Zeo-GmCYP1. For subcellular localization study, the pDONR-Zeo-GmCYP1 was recombined with the destination vector pEarlyGate101^65^ using the LR clonase reaction mix (Invitrogen, USA). The LR reaction was transformed into *E. coli* DH5α, PCR screened, then transformed into *Agrobacterium tumefaciens* GV3101 for plant transformation.

For *in planta* protein-protein interaction study, pDONR-Zeo-GmCYP1 was recombined separately with pEarlyGate201-YN and pEarlyGate202-YC to obtain pEG201-GmCYP1-YN and pEG202-GmCYP1-YC, respectively. The recombinant plasmids were transformed into *E. coli* DH5*α,* PCR screened, and then transformed into *A. tumefacians* GV3101. For FRET analysis, pDONR221-GmMYB176 and pDONR-Zeo-GmCYP1 were recombined into the pEG101 or pEG102 upstream of the YFP or CFP sequence under the control of the CaMV 35 S promoter. The recombinant plasmids were transformed into *E. coli* DH5α, PCR screened, and then transformed into *A. tumefacians* GV3101.

The promoter fragment of *GmCYP1* (1148 bp) was amplified using the primers GmCYP1-P-F: 5′-GGGGACAAGTTTGTACAAAAAAGCAGGCTCATCGTACTGCGATTTGAACGCAAGACTTC-3′, and GmCYP1-P-R: 5′- GGGGACCACTTTGTACAAGAAAGCTGGGTAGTTGCTGCAGAGAAGAGAAGCGTAAATGAC-3′, cloned into pDONR-Zeo (Invitrogen, USA), as described previously, followed by the recombination in the destination vector pMDC162 to obtain pGmCYP1pro-GUS. The pGmCYP1pro-GUS was transformed into *A. tumefaciens* GV3101 by electroporation, and then into wild-type *Arabidopsis* Col-0 by floral dip method^66^.

### Subcellular localization and bimolecular fluorescent complementation assay

The subcellular localization of GmCYP1 was studied by infiltrating *A. tumefaciens* GV3101 carrying pEG101-GmCYP1 into *N. benthamiana* leaves, as described by Sparkes *et al*.^67^. For co-expression, equal volumes of two construct-bearing strains, suspended in Gamborg’s solution, were mixed together and then infiltrated into *N. benthamiana* leaf epidermal cells. The protein expression was visualized by confocal microscopy using a Leica TCS SP2 inverted confocal microscope. An excitation wavelength of 514 nm was used for YFP imaging, and 525–545 nm emissions were collected. For visualization of CFP, an excitation wavelength of 458 nm was used, and emissions were collected between 465–495 nm.

### Fluorescence resonance energy transfer (FRET) assay

Equal volumes of two construct-bearing strains containing YFP and CFP fusions (in Gamborg’s solution), were mixed together and then infiltrated into *N. benthamiana* leaf epidermal cells. The protein expression was visualized by confocal microscopy using a Leica TCS SP2 inverted confocal microscope. An excitation wavelength of 458 nm and 514 nm were used for CFP and YFP imaging respectively. FRET acceptor bleaching, with CFP as donor and YFP as acceptor, was carried out by following Leica confocal application manual. The average of the FRET efficiency was calculated from multiple samples (n > 15).

### Histochemical GUS assay

For histochemical GUS staining, T_2_ transgenic *Arabidopsis* tissues were used. Tissues were incubated in dark in a solution containing 100 mM sodium phosphate buffer pH 7.0, 10 mM EDTA, 0.05% Triton X-100, 1 mM potassium ferricyanide, 1 mM potassium ferrocyanide and 1 mM X-Gluc (5-Bromo-4-chloro-3-indolyl-β-D-glucuronide) for 16 h at 37 °C with gentle shaking. De-staining was carried out with 95% ethanol for 4 to 6 times. A LeicaM2 FLIII^TM^ microscope with a QImaging Retiga 2000 R camera was used to take the photographs. For staining of silique and seeds, improved clearing method was used^68^.

### Yeast two-hybrid assay

Yeast two-hybrid assay (Y2H) was performed using the Matchmaker® Gold Two-Hybrid System (Clontech Laboratories, Inc., USA). Briefly, GmMYB176 was cloned into the vector pGBKT7 as the bait. The cDNA library as prey was generated by SMART™ cDNA Synthesis technology (Clontech Laboratories, Inc., USA) from soybean embryos (50–60 days after pollination) and fused to GAL4 activation domain. Screening was performed by co-transformation of bait and prey using Mate & Plate^TM^ library system (Clontech Laboratories, Inc., USA). After transformation, yeast cells were spread on SD/Ade/-His/-Leu/-Trp agar plates, and incubated at 30 °C for 5 days.

## Additional Information

**How to cite this article**: Mainali, H. R. *et al*. Soybean cyclophilin GmCYP1 interacts with an isoflavonoid regulator GmMYB176. *Sci. Rep.* **7**, 39550; doi: 10.1038/srep39550 (2017).

**Publisher's note:** Springer Nature remains neutral with regard to jurisdictional claims in published maps and institutional affiliations.

## Supplementary Material

Supplementary Tables
