# Supplementary material for: Soybean cyclophilin GmCYP1 interacts with an isoflavonoid regulator GmMYB176
Source: Sci Rep. 2017 Jan 11;7:39550. doi: 10.1038/srep39550 (PMC5225424; doi:10.1038/srep39550)
Supplement: Supplementary Tables [file srep39550-s1.doc]

**Soybean cyclophilin GmCYP1 interacts with an isoflavonoid regulator GmMYB176**

**Hemanta Raj Mainali, Arun Kumaran Anguraj Vadivel, Xuyan Li, Mark Gijzen and Sangeeta Dhaubhadel**

**Table S1** List of sequences used for the multiple sequence alignment with GmCYP1

| **Name** | **Species** | **Accession #** | **AA Identity (%)** | **AA Similarity (%)** |
| --- | --- | --- | --- | --- |
| GhCYP1 | *Gossypium hirsutum* | ACT63839.1 | 91 | 96 |
| ROC3 | *Arabidopsis thaliana* | NP_179251.1 | 88 | 94 |
| ROC6 | *Arabidopsis thaliana* | NP_179709.1 | 84 | 92 |
| ROC1 | *Arabidopsis thaliana* | AAB71402.1 | 84 | 92 |
| BnCYP | *Brassica napus* | AAA62706.1 | 83 | 91 |
| ROC5 | *Arabidopsis thaliana* | NP_195213.1 | 79 | 87 |
| CcCYP1 | *Cajanus cajan* | ADB04247.1 | 73 | 81 |
| hCYP-D | *Homo sapiens* | pdb|2Z6W|A | 71 | 80 |
| hCYP-A | *Homo sapiens* | NP_066953.1 | 69 | 79 |
| AtCYP63 | *Arabidopsis thaliana* | NP_191899.1 | 67 | 73 |
| AtCYP40 | *Arabidopsis thaliana* | NP_565381.1 | 62 | 73 |
| Cpr1 | *Saccharomyces cerevisiae* | NP_010439.1 | 63 | 74 |

AA, Amino acid

**Table S2** Sequence of oligonucleotides used for qPCR of soybean genes in AgNO_3_ treated and control tissues.

| **Gene** | **Primer Name** | **Sequence** | **Amplicon Size (bp)** |
| --- | --- | --- | --- |
| ***GmCYP1*** | qGmCYP1-F | 5'- CGTTGTCATCGCCAACTGC -3' | 145 |
|  | qGmCYP1-R1 | 5'- CACAATCCCCTAAACGACGACAC -3' |  |
| ***GmIFS2*** | qIFS2F | 5'- GGGTCATCGTCATCATCATCATATG -3' | 237 |
|  | qIFS2R | 5'- GGGGATAAATGATGTGGCAAC-3' |  |
| ***GmCHS8*** | CHS8/qRTf1 | 5’- GCTCCCATTTAATTGATTTCTGAA -3’ | 245 |
|  | CHS78/2EXNr | 5’- GACTTGTCACACATGCGCTGGAA -3’ |  |
| ***GmCHI1B1*** | CHI1B1F | 5’- CTGAGAGTTGCAGCAGTTGCC -3’ | 165 |
|  | CHI1B1R | 5’- AACTGGTTTGCTCGTTTATTAAATGGG -3’ |  |
|  | PT01-QF1 | 5’- CGGCAATGTGCATGGTTTCA-3’ | 151 |
| ***GmPT*** | PT01-QR1 | 5’-TTGCCAATGCCATACCCACA-3' |  |
| ***CON4*** | CON4F | 5’-GATCAGCAATTATGCACAACG-3' | 106 |
|  | CON4R | 5’-CCGCCACCATTCAGATTATGT-3’ |  |
